# Nanotip-assisted photoreduction of silver nanostructures on chemically patterned ferroelectric crystals for surface enhanced Raman scattering

**DOI:** 10.1038/s41598-019-47523-8

**Published:** 2019-07-29

**Authors:** Tzyy-Jiann Wang, Hsuan-Wei Chang, Ji-Sheng Chen, Hai-Pang Chiang

**Affiliations:** 1 Institute of Electro-Optical Engineering, National Taipei University of Technology, Taipei, 10608 Taiwan; 2 Institute of Optoelectronic Sciences, National Taiwan Ocean University, Keelung, 20224 Taiwan

**Keywords:** Nanoparticles, Raman spectroscopy, Optical sensors

## Abstract

Nanotips made of metal and semiconductor have been widely utilized in versatile applications to strengthen the electric field through lightning rod effect and localized surface plasmon resonance (LSPR) effect. Here, we present the utilization of ferroelectric nanotips to assist photoreduction of silver nanostructures for surface enhanced Raman scattering (SERS). Ferroelectric nanotips with spontaneous polarization posses the unique feature of producing the permanent electrostatic field without requiring external excitation, which differs from the present nanotips requiring electrical and optical excitation. The enhanced electrostatic field promotes the formation of silver nanoparticles by reducing the effect of Stern layer and accelerating the movement of photoelectrons and silver ions to the template surface. Experimental results show that sharp ferroelectric nanotips facilitate the formation of large-diameter nanoparticles with strong LSPR action. Compared to the conventional ferroelectric templates, the SERS substrates using nanotip-equipped ferroelectric templates produce 5.51 times larger Raman intensity, which can be further increased by >10.76 times by increasing the reaction time. The proposed SERS substrate owns the limit of detection <10^−8^ M and the enhancement factor of 2.3 × 10^9^. The presented ferroelectric nanotips with permanent electrostatic field would open promising applications in the versatile areas, such as nanomaterial fabrication and optoelectronic devices.

## Introduction

Ferroelectric lithography is an emerging technique to produce micro-/nano-scale patterns correlating with the polarization distribution on the ferroelectric templates^1^. Ferroelectric materials have spontaneous polarization, which can be reversed in direction by applying the external electric field (E > E_C_, E_C_: coercive field) on the ferroelectric materials^2^. Ferroelectric templates are formed by selectively manipulating the polarization distribution on the ferroelectric substrate using scanning-probe-based techniques^3^ or electric poling in combination with optical lithography^4–7^. Control of polarization direction or intensity causes the changes of surface bound charge, near-surface band bending, and electrostatic field distribution^1^. Polarization dependent chemical reactivity on the ferroelectric templates has been explored for diverse applications, including photoreduction of metal nanostructures^4–11^, selective molecular adsorption and desorption^12,13^, and selective suppression of ice nucleation^14^. Among them, the photoreduction of metal nanostructures has received great interest due to the features of simple fabrication, time effectiveness, and reusable template. It has been applied in the fields of surface enhanced Raman scattering (SERS)^6–8,11^, plasmon enhanced optical gain^15^, and tunable wettability^16^.

Lithium niobate (LiNbO_3_) with large spontaneous polarization (P_S_ = 78 µC/cm^2^) and low density of near surface defect (d = 10^12^ cm^−2^) is a favorable material for ferroelectric template fabrication. Periodically modulating the spontaneous polarization in LiNbO_3_ by electric poling^4–7^ and chemical patterning^8,9^, such as proton exchange (PE), can produce periodically poled LiNbO_3_ (PPLN) templates and periodically proton-exchanged (PPE) LiNbO_3_ templates, respectively. The silver nanopattern arrays produced by the PPE template create a stronger SERS signal relative to the PPLN template^17^. The effects of photoreduction parameters^4–6,9,11^ (wavelength, polarization, intensity, illumination time, solution concentration, and electrostatic field) on the silver nanostructure, have been studied. Besides, other noble metal nanoparticles, such as gold, have been demonstrated by using PPLN templates^7^ and PPE templates^10^.

Nanotips made of metal and semiconductor have been produced on the substrates and star nanoparticles by various fabrication methods, such as micro-electrodischarge^18^, focused ion beam (FIB) milling^19^, electron cyclotron resonance (ECR) etching^20^, electrochemical etching^21^, chemical reduction^22^, vapor phase epitaxy^23^ and so on. They have been utilized in the diverse applications, such as scanning probing measurement^24^, nanoscale manipulation^25^, field emission devices^26^, tip enhanced Raman spectroscopy^27^, and surface enhanced Raman scattering^22^. Besides the nanoscale mechanical contact, the action of nanotips includes the enhancement of electric field by lightning rod effect and localized surface plasmon resonance (LSPR) effect under the electrical and optical excitation. Lightning rod effect produces the field enhancement by strong potential gradient caused by the curvature of metallic tip end^28^. The enhancement level is related to the tip radius, the cone angle, and the tip/sample distance^29^. For the LSPR effect, the collective oscillation of free electrons induced by optical excitation greatly enhances the localized electric field intensity around the nanostructure. The action of LSPR effect is the main mechanism of the electromagnetic enhancement of the SERS signal^30^. The enhancement factor (EF) is expressed as^31^:

1 $$EF({\lambda }_{S})=\frac{|{E}_{out}(\lambda ){|}^{2}\cdot |{E}_{out}({\lambda }_{S}){|}^{2}}{|{E}_{0}{|}^{4}}\approx \frac{|{E}_{out}({\lambda }_{S}){|}^{4}}{|{E}_{0}{|}^{4}}$$

where *E*_*out*_(*λ*) and *E*_*out*_(*λ*_*S*_) are the local electric field at the incident wavelength *λ* and the Stokes-shifted wavelength *λ*_*S*_, and *E*_0_ is the incident electric field. In general, the wavelength difference between *λ* and *λ*_*S*_ is much smaller than the linewidth of the surface plasmon mode, thus *E*_*out*_(*λ*) is approximately equal to *E*_*out*_(*λ*_*S*_) and this approximation results in the third item in Eq. 1. The proportionality of EF to the fourth power of the localized electric field intensity greatly enhances the SERS signal intensity.

In this work, we demonstrate the nanotip-enhanced photoreduction of silver nanoparticle array by using PPE templates. Ferroelectric nanotips with spontaneous polarization can produce the permanent electrostatic field without requiring external excitation, which differs from metal nanotips and semiconductor nanotips requiring electrical and optical excitation to produce the electric field. To the best of our knowledge, the characteristics of ferroelectric nanotips have not been studied and their applications remain to be explored. The enhancement of electrostatic field by ferroelectric nanotips is demonstrated by the simulation using the finite element method and the experimental measurement using the electrostatic force microscopy (EFM). The promotion of photoreduction reaction causes the increase of nanoparticle diameter and distribution density, and thus enhances the Raman signal intensity. The dependence of nanoparticle properties and the SERS enhancement degree on the production parameters is also discussed.

## Results

**Figure 1:**
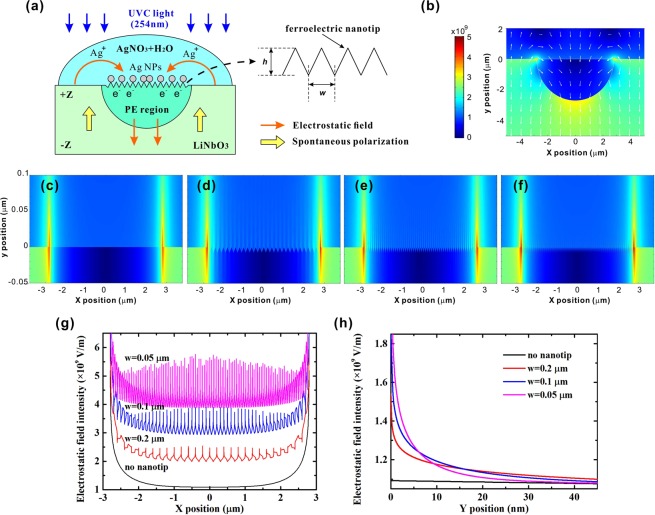
(**a**) Schematic diagram of the photoreduction process using the nanotip-equipped PPE templates. Inlet: simulated nanotip structure. Intensity and vectorial distribution of electrostatic field (**b**) around the ferroelectric template without chemical etching; and (**c**–**f**) around the surface of the PE regions (**c**) without chemical etching; and with the nanotips of width (**d**) 0.2 μm; (**e**) 0.1 μm; (**f**) 0.05 μm. Electrostatic field profiles (**g**) along the X direction; (**h**) along the Y direction, near the nanotip top on the PPE templates.

The schematic diagram of photoreduction process using the nanotip-equipped PPE template is shown in Fig. 1(a). The PE region has a semi-elliptical shape according the experimental result. Periodically modulating spontaneous polarization of LiNbO_3_ by PE produces the positive/negative surface bound charges and the permanent electrostatic field. The ferroelectric nanotips, which emit the enhanced permanent electrostatic field, are produced in the regions with the opening of the PE mask by chemically etching the proton-exchanged LiNbO_3_. When the UVC light (photon energy ~4.89 eV) is illuminated on LiNbO_3_ (bandgap ~3.9 eV), the photoelectrons (negative charge) are excited by the photons and then are accelerated by the electrostatic field toward the surface of PE region. In the AgNO_3_ solution, the silver ions (positive charge) are also accelerated by the electrostatic field toward the surface of PE region and then are reduced by photoelectrons to form silver nanoparticle array in this photoreduction process. Because the EFM output signal intensity is proportional to the gradient of electrostatic field intensity, the enhancement degree of electrostatic field produced by ferroelectric nanotips can not be measured directly. The electrostatic field intensity in the PPE template is calculated using the finite element method by solving the Gauss’s law and the generalized constitutive relation. The nanotip structure is approximated by a triangular shape with height *h* and width *w*, as shown in the inlet of Fig. 1(a). The intensity and vectorial distribution of electrostatic field in the PPE template without chemical etching is displayed in Fig. 1(b). The directions of electrostatic field inside and outside the PPE template are obviously different, which is the same as expected in Fig. 1(a). The electrostatic field above the PPE template is directed from the non-PE region to the PE region. Inside the PPE template, the electrostatic field has a downward direction. The edges on both sides of PE region have rather strong electrostatic field, by which more silver ions and more photoelectrons are concentrated on the solution/template interface and cause the formation of larger nanoparticles or even nanowires at these places.

**Figure 2:**
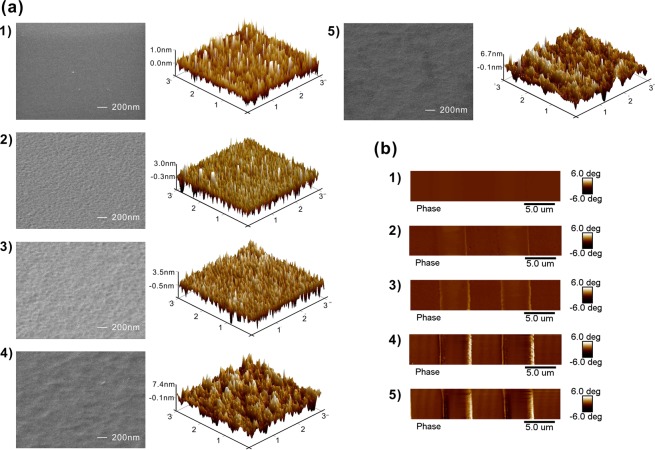
(**a**) Top view (left) and 3D view (right) of the AFM images and (**b**) the EFM images; of the PPE templates produced with the chemical etching time (1) 0 min (no etching); (2) 1 min; (3) 2 min; (4) 3 min; (5) 4 min.

The enhancement degree of electrostatic field by nanotips is related to their geometrical structure. In order to understand the effect of nanotip shape on the electrostatic field distribution near the surface of PE region, the enlargement of the electrostatic field distribution in the PPE templates without and with chemical etching are presented in Fig. 1(c–f). In the simulation, the nanotips have a height of 7.45 nm, which is the average height calculated from the maximal roughness of 2.15 nm measured in experiments. The nanotip widths under consideration are *w* = 0.05, 0.1, and 0.2 μm because the nanotip widths in the AFM images in Fig. 2 are distributed in the range of 0.05 μm~0.2 μm. For the PE region without chemical etching (Fig. 1(c)), the electrostatic field at the edges of PE region has strong intensity and can be extended to a distance of 200 nm toward the upper side. Above the most surface of PE region, the electrostatic field is rather weak and its extension distance is short. For the templates with the nanotips, the observation of the electrostatic field distribution in Fig. 1(d–f) shows that the electrostatic field at the edges of PE region still has strong intensity and the same extension distance, which are not affected by the existence of nanotips. The comparisons of electrostatic field profiles near the nanotip top along the lateral and longitudinal directions are shown in Fig. 1(g,h). It is noted that when the nanotip width decreases from 0.2 μm to 0.05 μm, the electrostatic field intensity is obviously enhanced but the strong-field extension range is shortened. The field extension range is around 25 nm for the PPE templates with ferroelectric nanotips, which is similar to the cases for metal nanotips^29^. The peak intensities of electrostatic field for the PPE templates without nanotips and with nanotips of width 0.2, 0.1, 0.05 μm are 1.160 × 10^9^, 1.541 × 10^9^, 1.900 × 10^9^ and 2.761 × 10^9^ V/m, respectively. It is noted that the enhancement of electrostatic field by the nanotips of width 0.05 μm is as high as 2.38 times in comparison with that without nanotips. The field enhancement by shrinking the nanotip width is due to the decrease of the radius of nanotip end, which is the same as the metal tips with applying external voltage^29^. Further increase of nanotip height or reduction of nanotip width will form the nanotips with smaller tip radius and produce the stronger electrostatic field on the top of nanotips.

**Figure 3:**
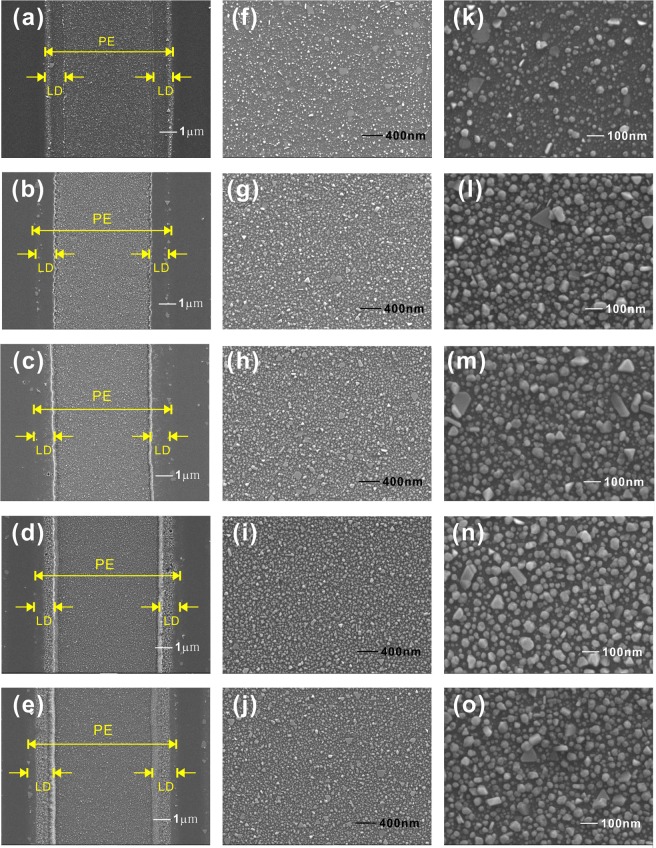
SEM images of Ag nanoparticles photoreduced on the PPE templates produced with the chemical etching time (**a**,**f**,**k**) 0 min (no etching); (**b**,**g**,**l**) 1 min; (**c**,**h**,**m**) 2 min; (**d**,**i**,**n**) 3 min; (**e**,**j**,**o**) 4 min. with the magnification (**a**–**e**) 10 K; (**f**–**j**) 30 K; (**k**–**o**) 100 K. (PE: proton-exchanged region; LD: proton lateral-diffusion region).

**Figure 4:**
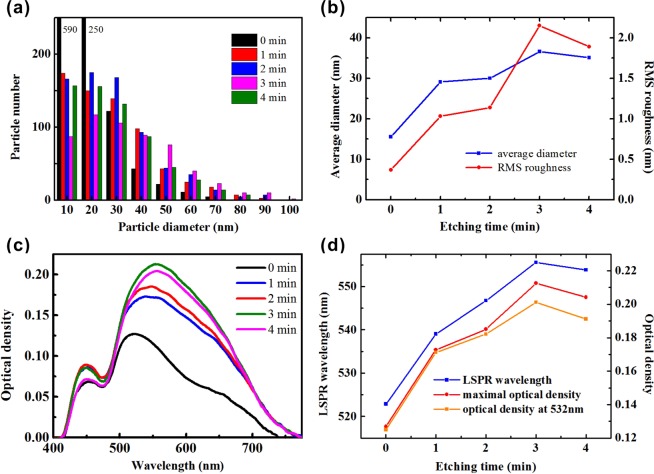
(**a**) Distribution of silver nanoparticle diameter and (**c**) absorption spectra of SERS substrates produced with the chemical etching time 0, 1, 2, 3, 4 min. Dependence of (**b**) average nanoparticle diameter and RMS roughness; and (**d**) LSPR wavelength, maximal optical density, and optical density at 532 nm; on the chemical etching time.

**Figure 5:**
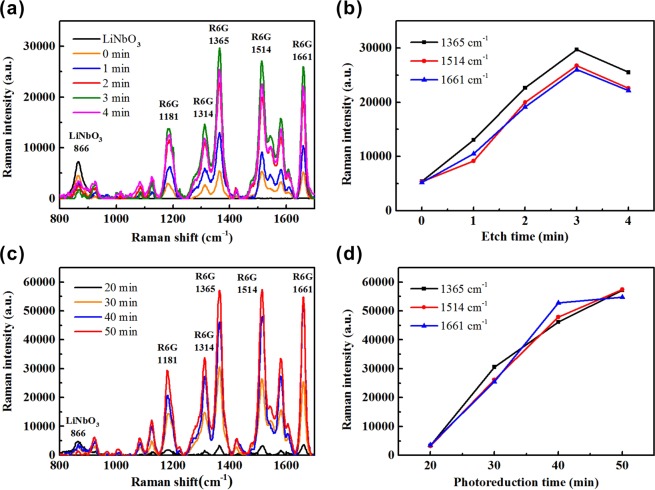
Raman spectra of R6G molecules on the SERS substrates produced with (**a**) the chemical etching time 0, 1, 2, 3, 4 min; and (**c**) the photoreduction time 20, 30, 40, 50 min. Dependence of Raman intensity at the peak 1365, 1514, 1661 cm^−1^ on (**b**) the chemical etching time; (**d**) the photoreduction time.

In order to characterize the ferroelectric nanotips, the PE regions without and with chemical etching are measured by atomic force microscopy (AFM) and electrostatic force microscopy (EFM) to manifest their surface and electrical properties. The observation of AFM images in Fig. 2(a) shows that when the chemical etching time increases from 0 min to 3 min, the surface morphology of PE regions becomes rougher, which make the nanotip height increase. The extension of etching time to 4 min causes somewhat reduction of nanotip height. The nanotip height does not increase monotonically with the etching time due to the maskless etching process. Excessive etching time causes the top of nanotips to be quickly etched and reduces the overall nanotip height. The nanotip structure can be obviously observed in Fig. 2(a-4) and 2(a-5). The root-mean-squared (RMS) roughness for the etching time *t* = 0, 1, 2, 3, 4 min is 0.37 nm, 1.03 nm, 1.14 nm, 2.15 nm, and 1.89 nm. The sample for *t* = 3 min has the maximal roughness of 2.15 nm and thus the largest average nanotip height. The electrostatic properties of PPE templates without and with chemical etching are shown in Fig. 2(b). The measured signal of EFM is the phase shift between the signal of probe actuation and the laser signal reflected from cantilever^32^ and is used as an indicator of electrostatic field intensity. The bright lines on two sides of PE region correspond to the strong electrostatic field at the edges of PE region, as shown in Fig. 1(c–f). The variation of EFM signal intensity in the PE region with the etching time is the same as that of RMS roughness. It is inferred that the increase of nanotip height facilitates the enhancement of electrostatic field intensity.

The SEM images of silver nanoparticles photoreduced using PPE templates produced with different chemical etching time are shown in Fig. 3. The silver nanoparticles are mainly grown in the PE region. For the sample without etching (*t* = 0 min), the number of silver nanoparticles is few and most of them have a small diameter, as shown in Fig. 3(a,f,k). Two thick lines on two sides of PE region in Fig. 3(a) correspond to the regions with larger silver nanoparticles, which are formed due to the strong electrostatic field at the edges of PE region. When the chemical etching time increases, the size of silver nanoparticles obviously grows and the number of large-diameter silver nanoparticles becomes larger. For the samples with chemical etching, two thick lines corresponding to the regions with larger nanoparticles shift toward the center of PE region. It is due to that the chemical etching proceeds on the region with the opening of the PE mask. The edges of unetched regions have a height larger than those of etched regions. The edge effect, which is similar to the tip effect, produces the enhanced electrostatic field and facilitates the formation of larger nanoparticles. The regions between the thick line and the outmost light line correspond to the regions of proton lateral-diffusion, as shown in Fig. 3(b–e). The observation of the SEM images in Fig. 3(k–o) shows that the photoreduced silver nanoparticles have various appearances, including polyhedrons, plates, triangles, rods, and irregular shapes.

The distribution of silver nanoparticle diameter for different etching time is displayed in Fig. 4(a). As expected, the PPE template without chemical etching mainly produces small nanoparticles with the diameter <20 nm. As the etching time increases, the electrostatic field enhanced by ferroelectric nanotips increases the numbers of photoelectrons and silver ions, and thus produces more large-diameter nanoparticles. It is noted that the PPE template for *t* = 3 min, which has the highest nanotips, possesses the maximal number of nanoparticles in the large diameter range of 50~90 nm in comparison with the other PPE templates. The variation of the average nanoparticle diameter and the RMS roughness with the etching time is shown in Fig. 4(b). Both of them have the same trend with the etching time. It is inferred that the electrostatic field enhanced by ferroelectric nanotips can effectively increase the growth rate of silver nanoparticles and facilitates the formation of large-diameter nanoparticles.

The PPE templates with photoreduced silver nanoparticles are used as SERS substrates. When silver nanoparticles are excited by the incident lightwave, the LSPR phenomenon occurs accompanying with the optical absorption at the specific wavelength and the localized surface plasma wave induced around the nanoparticles. The theoretical extinction spectrum of metal nanoparticles caused by LSPR is expressed as^31^:

2 $$I(\lambda )=\frac{18\pi \cdot {\varepsilon }_{out}^{3/2}\cdot V\cdot N}{\lambda \cdot \,\mathrm{ln}(10)}[\frac{{\varepsilon }_{i}(\lambda )}{{({\varepsilon }_{r}(\lambda )+\chi \cdot {\varepsilon }_{out})}^{2}+{\varepsilon }_{i}^{2}(\lambda )}]$$

where *ε*_*out*_ is the dielectric constant of the surrounding environment, *ε*_*r*_ and *ε*_*i*_ are the real and imaginary components of the metal dielectric function, *V* is the nanoparticle volume, *N* is the number of polarizable elements in the nanoparticle, and χ is the geometric factor with the value of 2 for the sphere. This equation describes the effects of nanoparticle volume and geometric shape on the optical absorption. The absorption spectra of the SERS substrates produced with different etching time are shown in Fig. 4(c). The absorption spectra exhibit two main absorption peaks. The first peak at around 450 nm is almost independent of the chemical etching time and is attributed to the nanoparticle seeds with diameter around 10 nm. The second one is variable in the range from 520–560 nm as the chemical etching time increases. The main absorption wavelength called LSPR wavelength corresponds to the wavelength with the maximal optical density. For the SERS substrate without chemical etching, the LSPR wavelength appears at 522.9 nm. When the etching time increases from 0 min to 1 min, the obvious increase of optical absorption means that the nanotip formation effectively enhances the production of silver nanoparticles. The dependences of LSPR wavelength, maximal optical density, and optical density at 532 nm, on the etching time are shown in Fig. 4(d). As the etching time increases from 0 min to 4 min, the LSPR wavelength initially increase at a steady rate and then slightly drops. Because larger silver nanoparticles own longer LSPR wavelengths, the increase of LSPR wavelength with the etching time represents the formation of larger nanoparticles in the templates with higher nanotips, which is consistent with the result presented in Fig. 4(b). The same trend is observed for the maximal optical density. The raise of maximal optical density represents the increase of number of nanoparticles excited by the lightwave at the LSPR wavelength. Hence, when the etching time increases from 0 min to 3 min, the rise of nanotip height not only promotes the enlargement of nanoparticles but also the growth of number of nanoparticles with the LSPR action. The further increase of etching time causes the reduction of nanotip height and thus decreases the average nanoparticle diameter. Among five PPE templates, the PPE template produced with *t* = 3 min possesses the largest average nanoparticle diameter 36.6 nm. The maximal number of nanoparticles in the diameter range of 50~90 nm facilitates the strong optical absorption for the visible light. Hence, the PPE template produced with *t* = 3 min can produce the largest optical absorption and thus the strongest electric field enhancement by the LSPR excitation. Because higher nanotips facilitate the enhancement of electrostatic field, the nanotip fabrication using the anisotropic etching techniques in future is promising to strengthen the nanotip-assisted photoreduction. Since the subsequent Raman measurement is performed using the laser light at the wavelength of 532 nm, the optical absorption at 532 nm is essential for the effective LSPR excitation and the enhancement of electric field intensity around nanoparticles. It is noted that the variation of optical density at 532 nm with the etching time is similar to that of the maximal optical density. The larger difference between the optical density at 532 nm and the maximal optical density appears at the SERS substrates produced with *t* = 3 min and 4 min. It is due to that the LSPR wavelengths of these two cases have a larger deviation from 532 nm.

The SERS spectra of the R6G molecules on the LiNbO_3_ substrate and the SERS substrates produced with different etching time are shown in Fig. 5(a). The measured Raman peaks of the R6G molecules are mainly located at 1181 cm^−1^, 1314 cm^−1^, 1365 cm^−1^, 1514 cm^−1^ and 1661 cm^−1^. The R6G Raman peak at 1181 cm^−1^ and 1365 cm^−1^ are attributed to C-H_x_ in-plane bending and the COO- vibration^33^. Those at 1514 cm^−1^, and 1661 cm^−1^ are related the C-C ring stretching vibration^33^. The Raman peak of LiNbO_3_ is only observed at 866 cm^−1^, which can be easily separated from the Raman peaks of R6G molecules. As the etching time increases from 0 min to 4 min, the intensity of all Raman peaks of R6G molecules has similar variation trend that the Raman intensity initially increases at a larger rate and then is reduced slightly at last. Three Raman peaks at 1365 cm^−1^, 1514 cm^−1^, and 1661 cm^−1^ with stronger intensity are considered in subsequent comparisons and the dependences of their intensities on the etching time are presented in Fig. 5(b). For all these three Raman peaks, the SERS substrate produced with *t* = 3 min has the maximal Raman intensity. The strongest electric field enhancement results from a lot of closely adjacent, large-diameter silver nanoparticles, as indicated in Fig. 3(n), which possesses high density of nm-scale inter-particle gap and thus gap plasmon resonance. It is noted that the variation of the Raman intensity with the etching time for these three Raman peaks has almost the same slopes on two sides of the optimal etching time although three of them originate from different molecular modes. It represents that the formed silver nanoparticles have the uniform SERS enhancement action for different molecular modes. In comparison with the conventional SERS substrate without nanotips, the proposed nanotip-equipped SERS substrate produced with *t* = 3 min possess 5.51, 5.01, 4.98 times larger Raman intensities at 1365 cm^−1^, 1514 cm^−1^, and 1661 cm^−1^.

In order to understand the action time of ferroelectric nanotips during the photoreduction process, the dependence of the Raman intensity using nanotip-equipped SERS substrates on the photoreduction time is investigated. The Raman spectra of R6G molecules on the SERS substrates produced with the photoreduction time 20, 30, 40, 50 min, are shown in Fig. 5(c) and the dependences of their Raman intensity at 1365 cm^−1^, 1514 cm^−1^ and 1661 cm^−1^ on the photoreduction time are presented in Fig. 5(d). When the photoreduction time increases from 20 min to 50 min, the Raman intensity at all of the R6G Raman peaks continuously increases. The observation of Fig. 5(d) shows that the Raman intensity at 1365 cm^−1^, 1514 cm^−1^, and 1661 cm^−1^ increases almost linearly with the photoreduction time. In comparison with the conventional SERS substrate without nanotips, the Raman intensities at 1365 cm^−1^, 1514 cm^−1^ and 1661 cm^−1^ can be further enhanced by 10.59, 10.76, 10.48 times by using nanotip-equipped SERS substrate produced with the etching time 3 min and the photoreduction time 50 min. The above results show that the ferroelectric nanotips can continue to take effect in the photoreduction process with the action time beyond 50 min. Because the Raman intensity continuously increases with the photoreduction time, it is expected that the Raman intensity using the nanotip-equipped SERS substrate can be further enhanced by optimizing the process parameters.

**Figure 6:**
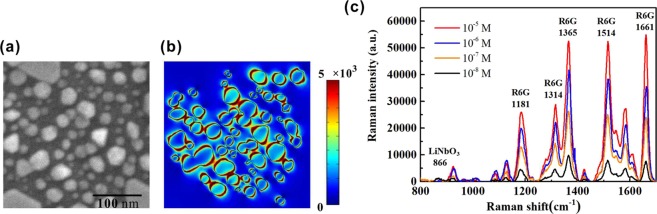
(**a**) SEM image of the silver nanoparticle array and (**b**) the simulated electric field distribution on the SERS substrate. The unit of electric field is V/m. (**c**) Raman spectra of the R6G molecules with the concentrations of 10^−5^ M, 10^−6^ M, 10^−7^ M, and 10^−8^ M on the SERS substrates produced with the etching time 3 min and the photoreduction time 50 min.

The performance of the proposed SERS substrate produced with the etching time 3 min and the photoreduction time 50 min is studied in detail. The SERS effect mainly arises from the electric field enhancement by the LSPR action in the nanostructure. We study the spatial distribution of electric field on the silver nanoparticle array on our SERS substrate by the computation using finite element software COMSOL. The SEM image of silver nanoparticle array in Fig. 6(a) is used to construct the simulation model^34^. Figure 6(b) presents the simulated electric field distribution excited by the incident light at 532 nm on the SERS substrate. It is found that the localized electric field around the nanoparticles with diameter >50 nm is stronger than that around the nanoparticles with smaller diameter. It is consistent with the experimental result that the PPE template for *t* = 3 min, which owns the maximal number of nanoparticles in the diameter range of 50~90 nm, has the strongest absorption at 532 nm. Besides, it also displays that there exists strong localized electric field in the separation between two nanoparticles. The strong electric field enhancement around the large-diameter nanoparticles and in the separation between two nanoparticles is supposed to be the main cause of the Raman signal enhancement in the proposed SERS substrate. In order to measure the limit of detection of the proposed SERS substrate, the Raman spectra for various concentrations of R6G solutions are measured. Figure 6(c) shows the Raman spectra of the R6G molecules with the concentrations of 10^−5^ M, 10^−6^ M, 10^−7^ M, and 10^−8^ M on the proposed SERS substrates. The Raman intensity at all the Raman peaks decreases with the descending R6G concentration with no exception. Because the measurement for the R6G solution of 10^−8^ M still has obvious Raman intensities at the Raman peak 1365 cm^−1^, 1514 cm^−1^, and 1661 cm^−1^, the proposed SERS substrate has the limit of detection as low as <10^−8^ M.

The enhancement level of Raman intensity of the proposed SERS substrate is quantitatively evaluated by calculating the enhancement factor, which is defined as follows:

3 $$EF=\frac{{I}_{SERS}/{C}_{SERS}}{{I}_{normal}/{C}_{normal}}=\frac{{I}_{SERS}}{{I}_{normal}}\cdot \frac{{C}_{normal}}{{C}_{SERS}}$$

where *I*_*SERS*_ and *C*_*SERS*_ are the Raman peak intensity and the concentration of probe molecule for the SERS measurement, *I*_*normal*_ and *C*_*normal*_ are the Raman peak intensity and the concentration of probe molecule for the regular Raman measurement. The SERS measurement was carried out with the 10^−8^ M R6G molecules adsorbed on the proposed SERS substrate. The regular Raman measurement was conducted with the 0.15 M R6G molecules adsorbed on the LiNbO_3_ substrate and its result is shown in Fig. S1 (See Supplementary Information). The EF calculation based on the Raman peak at 1514 cm^−1^ presents the EF value as high as 2.3 × 10^9^. The uniformity of the proposed SERS substrates is studied by measuring the Raman spectra at eight randomly selected spots on the silver nanoparticle array. The relative standard deviation (RSD) of the Raman intensity is less than 8.5% (See Supplementary Information). It demonstrates the uniform SERS enhancement on the proposed SERS substrate.

The nanotip-assisted photoreduction mechanism can be explained by considering the action of the electron flux and the ion flux. In the photoreduction process, the electrons generated by LiNbO_3_ under the above-bandgap illumination can reduce the silver ions to form silver nanoparticles by the reaction Ag^+^ + e^−^ → Ag^0^. The photon flux (photons/cm^2^·s) can be calculated from *J*_*ph*_ = (*I*_0_·*λ*)/(*h*·*c*), where *I*_0_ is the light intensity, *λ* is the wavelength, *h* is Planck’s constant, and *c* is the speed of light. The electron flux is smaller than the photon flux due to the light scattering by photoreduced metal nanoparticles, the lower efficiency of electron/hole pair generation, and the electron/hole recombination. In the aqueous solution, the ion flux density (ions/cm^2^·s) is calculated using the equation *J*_*ion*_ = *v*_0_·*C*_0_, where *v*_0_ is average velocity toward the template surface and *C*_0_ is the ion concentration near the template surface. This work prepares the SERS substrates using the illumination intensity *I*_0_ = 10 mW/cm^2^ and the 0.01 M AgNO_3_ solution, which correspond to *J*_*ph*_ = 1.28 × 10^16^ (photons/cm^2^·s) and *J*_*ion*_ = 1 × 10^22^ (ions/cm^2^·s) with the flux ratio *J*_*ion*_/*J*_*ph*_ = 7.83 × 10^5^. The ion flux is greater than the photon flux in this work. Periodically modulating the spontaneous polarization of LiNbO_3_ produces the positively charged area and the negatively charged areas on the template surface. In order to achieve an energetically stable state, the surface bound charges are partially screened by adsorption of charged molecules in the AgNO_3_ aqueous solution, which is commonly referred as electrical double layer or Stern layer^4,5^. On the photoreduction area, the crowding of NO_3_^−^ ions and polarized water molecules on the template surface decreases the availability of Ag^+^ for reduction reactions^9^. Due to the action of Stern layer, the deposition height increases roughly linearly with the photoreduction time and saturates at the height limited by the Debye length, which is 3.04 nm at the photoreduction time 15min^9^. For the nanotip-equipped SERS substrates, the electrostatic field enhanced by ferroelectric nanotips not only boost the NO_3_^−^ ions away from the photoreduction region but also attract the Ag^+^ ion to here. Besides, it also accelerates the photoelectrons to the photoreduction region. Hence, the three-fold action of ferroelectric nanotips can effectively promote the photoreduction process and grow the silver nanoparticles without the deposition height limited by the Stern layer, which is indicated by the linear increase of Raman intensity with the photoreduction time beyond 50 min.

## Conclusion

In summary, we have demonstrated the nanotip-assisted photoreduction of silver nanoparticles using ferroelectric nanotips with the permanent electrostatic field for surface enhanced Raman scattering. To the best of our knowledge, it is the first time to demonstrate the ferroelectric nanotips with the permanent electrostatic field and their applications. The electrostatic field emitted by ferroelectric nanotips can be enhanced by reducing the radius of nanotip end. In the nanotip-assisted photoreduction, the enhancement of electrostatic field facilitates the formation of large-diameter nanoparticles with strong LSPR action by reducing the effect of Stern layer and accelerating the movement of photoelectrons and silver ions to the template surface. In comparison with the conventional SERS substrates, the proposed SERS substrates with ferroelectric nanotips can enhance the Raman intensity by 5.51 times using the chemical etching for 3 min. The enhancement of Raman intensity by 10.76 times has been achieved by extending the photoreduction time. The action time of ferroelectric nanotips in the photoreduction process is confirmed to be longer than 50 min by the observation of continuous increase of Raman intensity with the photoreduction time. Optimization of the photoreduction parameters and modification of the ferroelectric nanotip shape by using anisotropic etching techniques will promote the nanoparticle formation and further enhance the Raman intensity. The proposed ferroelectric templates with nanotips have features of simple fabrication, efficient photoreduction, and high flexibility. The ferroelectric nanotips with the attractive feature of permanent electrostatic field without requiring external excitation are promising to open various novel applications in the versatile areas.

## Materials and Methods

### Fabrication

Ferroelectric templates are produced on z-cut, optical grade, congruent LiNbO_3_ substrates (Crystal Technology Inc.) with thickness 1 mm and size 1 cm × 1 cm. After cleaning by ultrasonic bath in acetone and methanol for 10 mins, the 120nm-thick Cr film patterned with width 5 μm and period 10 μm, which is used as PE mask, is formed on the +z face of LiNbO_3_ substrates by RF magnetron sputtering, optical lithography, and chemical etching with the Cr-7 etchant. For the production of PE regions, the samples are immersed in benzoic acid at 240 °C for 5hrs. The PE region has a semi-elliptical-shape of width 5.6 μm and depth 2.7 μm, which is the same as the one^8,10^ at 200 °C for 24hrs. Ferroelectric nanotips on the PPE regions are produced by immersing the samples in the HF solution with concentration 49% for 1, 2, 3 and 4 mins to form different nanotip height. After removing the Cr films and cleaning the substrate, the production of PPE templates with ferroelectric nanotips is finished.

### Photoreduction

The PPE templates are cleaned by ultrasonic bath in acetone and methanol for 10 mins and then by immersion in Piranha solution for 20 mins with afterward rinse under flowing de-ionized water for 10 mins. The template surface is dropped with the 0.01 M AgNO_3_ solution and then illuminated by the UVC light with the wavelength centered at 254 nm at a distance of 2 cm. After the specific photoreduction time, the templates are immersed in de-ionized water and then blown dry by nitrogen gas.

### AFM, EFM, and SEM measurements

Surface morphologies and surface electrostatic properties of ferroelectric templates are characterized by using an atomic force microscope (AFM, Bruker Dimension Icon) and an electrostatic force microscope (EFM, Bruker Dimension Icon). The AFM is performed at AC mode with the scan rate of 0.6 Hz using the highly doped silicon probe tip with a spring constant of 42 N/m and a resonant frequency of 330 kHz (Nanosensors). The EFM is operated using a tip voltage of 3 V and a lift height of 50 nm. Surface morphologies of silver nanoparticles on ferroelectric templates are obtained by using a field emission scanning electron microscope (FESEM, JEOL JSM7610F).

### Absorption and SERS measurement

In the absorption spectrum measurement, the light from the halogen light source is focused on the sample by a 10× objective lens. The transmitted light from the sample is collected by another 10× objective lens and subsequently coupled to the 100 µm-core-diameter optical fiber connected to the spectrometer (Ocean Optics, HR2000). In the SERS measurement, the 10^−5^ M solution of R6G dye used as a probe molecule is dropped on the sample surface for 2 hrs. The samples are then immersed in de-ionized water to remove unadsorbed dye molecules. The Raman spectrum measurements are carried out using a 532 nm DPSS laser and an Acton SP-2358 spectrometer equipped with TE-cooled CCD (Andor DV420A). A 50× objective lens (NA = 0.42) is used to focus the laser light on the target area and to collect the backward scattering light from the sample surface. The integration time of the Raman measurement is 5 sec.

## Supplementary information

Supplementary Information

## References

[1] Kalinin SV (2004). Ferroelectric Lithography of multicomponent nanostructures. Adv. Mater..

[2] Gopalan V, Mitchell TE, Sicakfus KE (1998). Switching kinetics of 180° domains in congruent LiNbO_3_ and LiTaO_3_ crystals. Solid State Commun..

[3] Liu X, Kitamura K, Terabe K, Hatano H, Ohashi N (2007). Photocatalytic nanoparticle deposition on LiNbO_3_ nanodomain patterns via photovoltaic effect. Appl. Phys. Lett..

[4] Sun Y, Nemanich RJ (2011). Photoinduced Ag deposition on periodically poled lithium niobate: Wavelength and polarization screening dependence. J. of Appl. Phys..

[5] Sun Y, Eller BS, Nemanich RJ (2011). Photoinduced Ag deposition on periodically poled lithium niobate: Concentration and intensity dependence. J. of Appl. Phys..

[6] Liu X (2013). Tunable and highly reproducible surface-enhanced Raman scattering substrates made from large-scale nanoparticle arrays based on periodically poled LiNbO_3_ templates. Sci. Technol. Adv. Mater..

[7] Liu X, Osada M, Kitamura K, Nagata T, Si D (2017). Ferroelectric-assisted gold nanoparticles array for centimeter-scale highly reproducible SERS substrates. Sci. Rep..

[8] Carville NC (2012). Photoreduction of SERS-active metallic nanostructures on chemically patterned ferroelectric crystals. ACS Nano.

[9] Carville NC, Manzo M, Denning D, Gallo K, Rodriguez BJ (2013). Growth mechanism of photoreduced silver nanostructures on periodically proton exchanged lithium niobate: Time and concentration dependence. J. Appl. Phys..

[10] Carville NC, Neumayer SM, Manzo M, Gallo K, Rodriguez BJ (2016). Biocompatible gold nanoparticle arrays photodeposited on periodically proton exchanged lithium niobate. ACS Biomater.-Sci. Eng..

[11] Wang T-J (2017). Electrostatic-field-tunable ferroelectric template for photoreduction of silver nanostructures applied in Raman scattering enhancement. Opt. Mater. Express.

[12] Zhang Z, Sharma P, Borca CN, Dowben PA, Gruverman A (2010). Polarization-specific adsorption of organic molecules on ferroelectric LiNbO_3_ surfaces. Appl. Phys. Lett..

[13] Li D (2008). Direct *in situ* determination of the polarization dependence of physisorption on ferroelectric surfaces. Nat. Mater..

[14] Ehre D, Lavert E, Lahav M, Lubomirsky I (2010). Water freezes differently on positively and negatively charged surfaces of pyroelectric materials. Science.

[15] Molina P (2016). Plasmon-assisted Nd^3+^-based solid-state nanolaser. Nano Letters.

[16] Al-Shammari RM, Manzo M, Gallo K, Rice JH, Rodriguez BJ (2017). Tunable wettability of ferroelectric lithium niobate surfaces: the role of engineered microstructure and tailored metallic nanostructures. J. Phys. Chem. C..

[17] Damm S (2012). Plasmon enhanced Raman from Ag nanopatterns made using periodically poled lithium niobate and periodically proton exchanged template methods. J. Phys. Chem. C..

[18] Jahan MP, Rahman M, Wong YS (2011). A review on the conventional and micro-electrodischarge machining of tungsten carbide. Int. J. Mach. Tools Manuf..

[19] Chen R, Hu K, Yu Y, Mirkin MV, Amemiya S (2016). Focused-ion-beam-milled carbon nanoelectrodes for scanning electrochemical microscopy. J. Electrochem. Soc..

[20] Chattopadhyay S, Lo HC, Hsu CH, Chen LC, Chen KH (2005). Surface-enhanced Raman spectroscopy using self-assembled silver nanoparticles on silicon nanotips. Chem. Mater..

[21] Silva EL, Silva RF, Zheludkevich M, Oliveira FJ (2014). Novel electrochemical method of fast and reproducible fabrication of metallic nanoelectrodes. Rev. Sci. Instrum..

[22] Lai CH (2017). Near infrared surface-enhanced Raman scattering based on star-shaped gold/silver nanoparticles and hyperbolic metamaterial. Sci. Rep..

[23] Park WI, Yi G-C, Kim M, Pennycook SJ (2002). ZnO nanoneedles grown vertically on Si substrates by non-catalytic vapor-phase epitaxy. Adv. Mater..

[24] Cano-Marquez AG (2015). Enhanced mechanical stability of gold nanotips through carbon nanocone encapsulation. Sci. Rep..

[25] Loplnski GP, Wayner DDM, Wolkow RA (2000). Self-directed growth of molecular nanostructures on silicon. Nature.

[26] Tao Cao (2016). Integrated ZnO Nano-electron-emitter with self-modulated parasitic tunneling field effect transistor at the surface of the p-Si/ZnO junction. Sci. Rep..

[27] Yano T (2013). Tip-enhanced nano-Raman analytical imaging of locally induced strain distribution in carbon nanotubes. Nat. Commun..

[28] Urbieta M (2018). Atomic-scale lightning rod effect in plasmonic picocavities: a classical view to a quantum effect. ACS Nano.

[29] Lenk S, Lenk C, Rangelow IW (2018). Theoretical investigation of the enhancement factor for a single field emitter in close proximity to the counter electrode. J. Vac. Sci. Technol. B.

[30] Saikin SK, Chu Y, Rappoport D, Crozier KB, Aspuru-Guzik A (2010). Separation of electromagnetic and chemical contributions to surface-enhanced Raman spectra on nanoengineered plasmonic substrates. J. Phys. Chem. Lett..

[31] Willets KA, Duyne RPV (2007). Localized surface plasmon resonance spectroscopy and sensing. Annu. Rev. Phys. Chem..

[32] Karatay DU, Harrison JS, Glaz MS, Giridharagopal R, Ginger DS (2016). Fast time-resolved electrostatic force microscopy: achieving sub-cycle time resolution. Rev. Sci. Instrum..

[33] Das G (2016). Few molecule SERS detection using nanolens based plasmonic nanostructure: application to point mutation detection. RSC Adv..

[34] Jie Z (2016). Ag-Cu nanoparticles encaptured by graphene with magnetron sputtering and cvd for surface-enhanced Raman scattering. Plasmonics.

