# Supplementary material for: Nanotip-assisted photoreduction of silver nanostructures on chemically patterned ferroelectric crystals for surface enhanced Raman scattering
Source: Sci Rep. 2019 Jul 29;9:10962. doi: 10.1038/s41598-019-47523-8 (PMC6662766; doi:10.1038/s41598-019-47523-8)
Supplement: Supplementary file 1 — Supplementary Information [file 41598_2019_47523_MOESM1_ESM.pdf]

Supplementary Information

Nanotip-assisted photoreduction of silver nanostructures on chemically patterned ferroelectric crystals for surface enhanced Raman scattering

Tzyy-Jiann Wang[1,*], Hsuan-Wei Chang[1], Ji-Sheng Chen[1], & Hai-Pang Chiang[2]

[1] *Institute of Electro-Optical Engineering, National Taipei University of Technology, Taipei 10608, Taiwan.*

[2] *Institute of Optoelectronic Sciences, National Taiwan Ocean University, Keelung, 20224, Taiwan.*

[*] *Correspondence to: f10939@ntut.edu.tw*

Measurement of enhancement factor

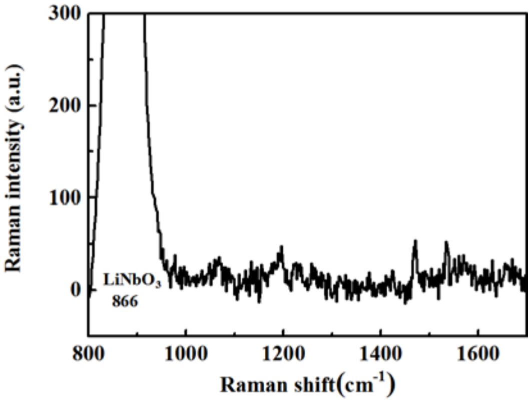

Figure S1 Raman spectra of the R6G molecules with the concentrations of 0.15 M on the $LiNbO_3$ substrates

Measurement of the uniformity of the SERS substrate

The uniformity of the proposed SERS substrates is studied by measuring the Raman spectra at eight randomly selected spots on the silver nanoparticle array. Figure S2 shows the Raman spectra of R6G molecules and their intensity distribution of Raman peaks at 1365 cm^{-1}, 1514 cm^{-1}, and 1661 cm^{-1} at eight randomly selected spots on the SERS substrate produced with the etching time 3min and the photoreduction time 50min. The values of relative standard deviation (RSD) of the Raman intensity at 1365 cm^{-1}, 1514 cm^{-1}, and 1661 cm^{-1} are 8.3%, 8.5%, and 7.6%. The intensity variation less than 8.5% demonstrates the uniform SERS enhancement on the proposed SERS substrate.

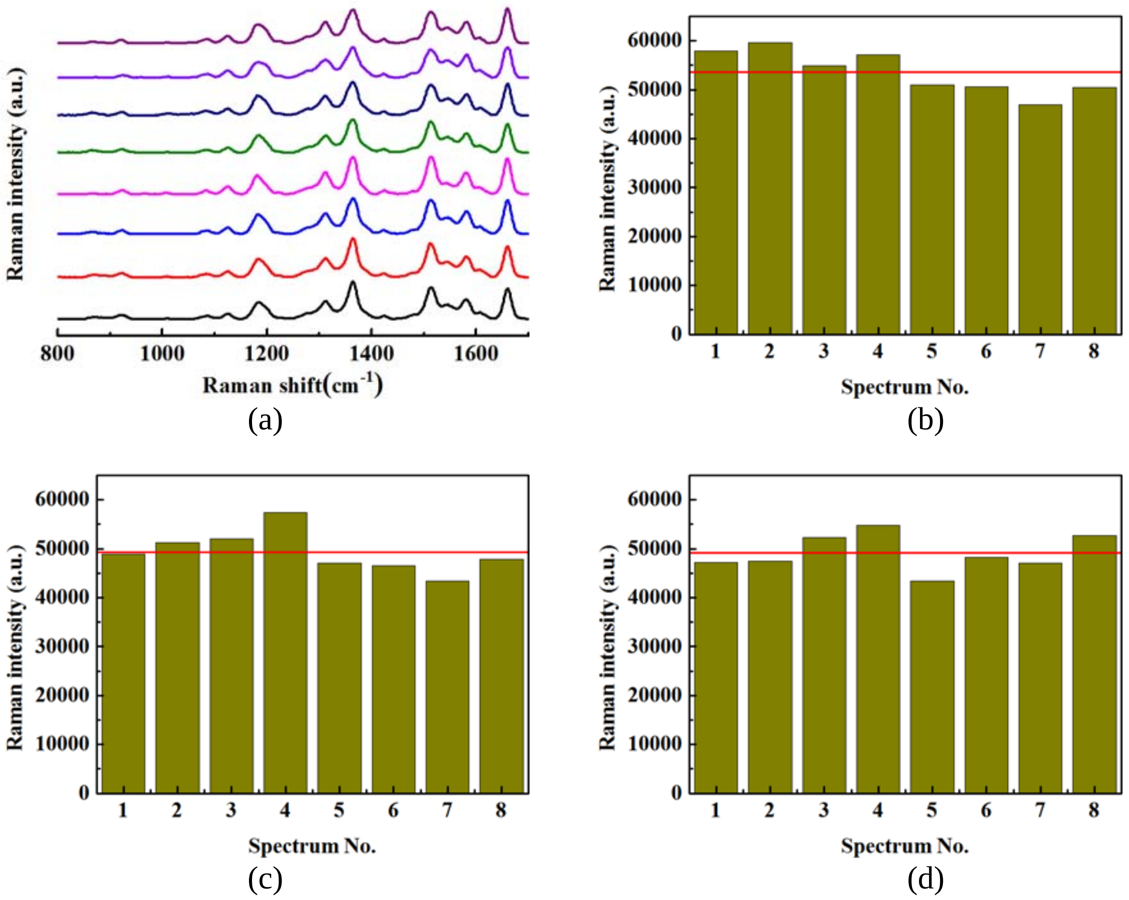

Figure S2 (a) Raman spectra of R6G molecules at eight randomly selected spots on the proposed SERS substrate; (b-d) Intensity distributions of Raman peaks at (b) 1365 cm^{-1}; (c) 1514 cm^{-1}; (d) 1661 cm^{-1} in eight Raman spectra.
